# VISTA as a prospective immune checkpoint in gynecological malignant tumors: A review of the literature

**DOI:** 10.1515/med-2023-0866

**Published:** 2023-12-11

**Authors:** Ran Ren, Xin Chang, Cong Chen, Hao Yu, Lu Han

**Affiliations:** Department of Graduate, Dalian Medical University, Dalian, 116044, China; Department of Gynecology, Dalian Women and Children’s Medical Group, Dalian, 116033, China; Laboratory of Early Diagnosis and Biotherapy of Malignant Tumors in Children and Women in Liaoning Province, Dalian, 116011, China

**Keywords:** VISTA, malignant tumors, tumor microenvironment, immunotherapy

## Abstract

V-domain Ig suppressor of T cell activation (VISTA), encoded by the human VSIR gene, is a B7 family checkpoint homologous to the programmed death-Ligand 1 sequence. In gynecologic malignancies, VISTA is abnormally expressed and regulates the tumor immune microenvironment, causing a high upregulation of VISTA expression in T-cells and myeloid cells in the tumor microenvironment and promoting tumor proliferation, progression, and immune tolerance. Here, we review the research progress of VISTA in ovarian, cervical, and endometrial cancers through its structure and immunomodulatory mechanism. The comprehensive study of VISTA is expected to improve the current problem of poor immunotherapeutic effects and provide new ideas for immune therapy in patients with gynecologic tumors.

## 1 Introduction

V-domain Ig suppressor of T cell activation (VISTA), also known as programmed death-1homolog (PD-1H) [1,2], is a type I transmembrane protein domain encoded by the human VSIR gene encoding a type I transmembrane protein subunit, a recently identified B7 family checkpoint with sequence homology to programmed death-Ligand 1 (PD-L1), and the highest similarity to PD-L1 [3,4]. Similar to PD-1 and cytotoxic T lymphocyte-associated antigen-4 (CTLA-4) immune checkpoints, VISTA decreases T cell activation and proliferation. VISTA is believed to exert significant immunological effects because of its specific structure, which can be both a ligand and a receptor. As a ligand, VISTA is expressed on antigen-presenting cells (APCs) and binds to receptors on T cells to inhibit downstream T cell activation. As a receptor, VISTA is expressed on T cells and transduces intracellular inhibitory signals on ligand binding to suppress T-cell activity [5]. In this review, we summarize the structure, expression, and immunomodulatory mechanism of VISTA, and suggest the feasibility of VISTA as a new immune checkpoint for the immunotherapy of gynecological tumors, to improve the treatment status and prognosis of gynecologic oncology patients.

## 2 Structure and expression of VISTA

The human VSIR gene encodes the VISTA protein. It is 279 amino acids in length, including a 162 amino acid extracellular structural domain, a 21 amino acid transmembrane structural domain, and a 96 amino acid cytoplasmic structural domain. The cytoplasmic structural domain lacks immunoreceptor tyrosine-based signal transduction sequences but contains multiple casein kinase two and phosphokinase C phosphorylation sites that play a significant role in signal transduction. VISTA gene sequence analysis revealed surprisingly high conservation, with 76% homology between mice and humans, especially in the cytoplasmic structural domain, making mice the most suitable experimental subjects for studying VISTA. The analysis of VISTA protein sequences revealed that VISTA is similar to the B7 family group of ligands (CD80, CD86, PD-L1, PD-L2, ICOSL, and CD276), all of which contain a conserved immunoglobulin variable-like fold. PD-L1 in the B7 family shares 23% sequence homology with VISTA [6]. However, unlike traditional B7 family members, the intracytoplasmic structural domain of VISTA does not contain ITAM, ITMM, or ITSM sequences. In contrast, the intracellular tail of VISTA contains two potential protein kinase C binding sites and a proline residue that may serve as a docking site and includes an Ig-V structural domain. Thus, VISTA may act as both a receptor and a ligand [2].

At the cellular level, VISTA is mainly found in the hematopoietic tissue of human peripheral blood mononuclear cells (PBMCs). VISTA was expressed at high levels on the CD11CloCD123+HLA-DR+lymphoid lineage and CD11C+CD123LOHLA-DR+ myeloid subpopulation of monocytes and dendritic cells, at moderate levels on CD4+T, CD8+T, and FOXP3+CD4+Tregs expressing cells, at low levels on thymocytes, CD56loNK cells, and completely absent on CD56hiNK cells and CD19+ B cells [7–9]. However, Bharaj has found that in human PBMCs, there was VISTA expression in CD3+ T cells and CD19+ B cells, which was considered as a different result of different antibody types [10]. In addition, the expression of VISTA gradually decreased with time *in vitro* culture.

At the tissues and organs, transcription of Vsir genes in adult individuals under normal conditions occurs mainly in lymphoid tissues such as the spleen, thymus, and bone marrow [11]. In non-hematopoietic organs, VISTA expression is usually highest in the brain, stomach, and thyroid, while it is expressed at intermediate levels in the spleen and liver and rarely in bone and heart [12]. Lower levels of VISTA-mRNA were detected in the lung, muscle, testis, kidney, and placenta.

A series of studies have examined VISTA expression in human tumors, including acute myeloid leukemia, prostate cancer, cutaneous melanoma, hepatocellular carcinoma, gestational trophoblastic tumors, and breast cancer [13–18]. Increased expression of VISTA in different tumors is usually accompanied by tumor progression and recurrence, and predicts low patient survival [19,20].

## 3 Immunomodulatory mechanisms of VISTA

When VISTA acts as a ligand, it negatively regulates T-cell activation [21]. The extracellular structural domain of VISTA interacts with coinhibitory receptors on T cells to inhibit T cell proliferation and cytokine (IL-2 and IFN-γ) production, and naïve T cells and memory T-cells are sensitive to Vista-mediated suppression by inhibiting phosphorylation of T cell receptor proximal signaling molecules (LAT, PLC-γ1, and SLP76) and upregulation of early activation markers (CD69, CD44, and CD25) [22]. In the presence of exogenous TGF-β, VISTA promotes the conversion of FOXP3-CD4+ T cells to FOXP3+ adaptive regulatory T cells. This effect contributes to the overall T-cell suppressive effect of VISTA.

When VISTA acts as a receptor, its expression on the surface of CD4+ T cells is independent of acting APCs on T cells. When CD4+ T cells were exposed to Vista–/–APCs, it resulted in increased cell proliferation and promoted the production of IFNγ, TNF-α, and IL-17A. It suggests that VISTA can act as a suppressor receptor for CD4+ T cells. Recently, Shahbaz et al. showed that Vista+ CD71+ red lineage cells produce more TGF-β and promote the production of Tregs from primitive CD4+ T cells by inhibiting p-Akt and p-mTOR [23].

Abnormal VISTA expression can cause sustained tumor growth acceleration; conversely, blocking VISTA expression enhances the T-cell antitumor response and slows tumor progression. A VISTA/PD-1 double knockout mouse model found that VISTA inhibits T cell activation through a pathway different from PD-1 to achieve immune escape of tumor cells (TCs) [22]. VISTA acts as a ligand on TCs and mediates negative immune regulation by inhibiting the activity of CD4+ and CD8+ T cells and suppressing immune cell (IC) activity. When VISTA molecules act as receptors, they are highly expressed in bone marrow-derived suppressor cells (MDSCs) and significantly inhibit IC responses in the tumor microenvironment (TME). VISTA expression is upregulated on MDSCs in various human tumor types, exerting a powerful tumor-infiltrating lymphocyte suppressing function and promoting their immunosuppression [24].

## 4 Association of VISTA with gynecologic tumors

As a close relative of PD-1/PD-L1, VISTA is also aberrantly expressed in gynecologic malignancies and regulates ICs and related cytokines in the TME, which promotes tumor proliferation, progression, and immune tolerance. VISTA expression has been demonstrated in tumor-associated ICs and/or TCs in different types of cancer. However, in contrast with its negative regulatory role in T-cell responses, increasing evidence suggests that VISTA expression on TCs or ICs correlates with improved survival among patients with ovarian, cervical cancer, and endometrial cancer.

### 4.1 Ovarian cancer and VISTA

Ovarian cancer has the highest mortality rate among the three primary female reproductive system tumors [25], and the current first-line treatment with surgery and postoperative platinum-based chemotherapy is ineffectual, with most patients experiencing postoperative recurrence and platinum resistance, which significantly affects the prognosis of ovarian cancer patients [26]. Recent immunotherapeutic approaches to kill ovarian cancer cells through CTLA-4, PD-1/PD-L1, and other immune target inhibitors such as Pembrolizumab and Nivolumab to interfere with immune modulation in ovarian cancer TME have shown initial success, and immunotherapy with VISTA and ovarian cancer is in full swing [27–29].

Mulati et al. have found that VISTA is expressed more often in ovarian cancer TCs than in normal cells. In mice with ovarian cancer cells expressing high levels of VISTA, a significant decrease in the abundance of CD8+ T cells in the TME, infiltration of IFN-γ-producing T cells, and upregulation of MDSC accumulation were observed [30]. This dynamic immunological alteration in the TME reflects accelerated migration of MDSCs to the tumor site and increased infiltration of immature myeloid cells that may differentiate into MDSCs at the tumor site. However, VISTA antibodies blocked the expression of VISTA on both TCs and ICs. However, the inhibitory effect of VISTA on tumors was much stronger than that on ICs. In a retrospective analysis of 65 ovarian cancer patients, the positive expression of VISTA was 30.8% in TCs and 44.6% in ICs. High expression of VISTA on TCs and ICs was correlated with advanced ovarian cancer and lymph node metastasis, probably because VISTA molecules can protect related TCs and ICs to achieve an immune escape effect [31].

Zong found by immunohistochemistry in 146 ovarian cancers that VISTA expression was associated with pathological type and PD-L1 expression. VISTA was frequently expressed in PD-L1-negative ovarian cancer tissues, and VISTA expression in TCs represented longer progression-free survival and a high survival rate [32]. This situation may be related to VISTA regulation of C10orf54 gene expression for tumor immune escape, but further in-depth study is needed.

In ovarian cancer mice animal experiments, the survival of mice added with anti-VISTA antibody was significantly prolonged. The expression of VISTA in TCs not only inhibited cytotoxic T lymphocytes (CTLs) from lysing antigen-specific cells but also considerably suppressed T cell proliferation. In addition, Mulati also found that VISTA treatment may be effective in ovarian cancer resistant to anti-PD-1 therapy expressing VISTA, which is expected to be a strategy to address PD-1 resistance in the future [30].

### 4.2 Cervical cancer and VISTA

Cervical cancer, one of the three major malignant tumors of the female reproductive system, has been greatly reduced by early screening, diagnosis, and treatment [33]. With the rapid development of surgery, radiotherapy, and chemotherapy, the treatment of cervical cancer has achieved specific achievements. However, the 5-year survival rate is still unsatisfactory, about 68%, and the recurrence rate for patients with advanced cervical cancer is about 40% [34]. Immunotherapy has a good prospect in the treatment of cervical cancer. Specific progress has been made through the treatment of PD-L1, CTLA-4, PARP, and other immune checkpoint inhibitors. However, it still has a limited role in advanced and recurrent cervical cancer, with high drug resistance, and usually requires combination therapy to be effective [35].

Human papillomavirus (HPV) positivity is a high-risk factor for the development of cervical cancer, and HPV positivity and viral load are independent risk factors for the recurrence of cervical cancer, which affects the outcome of cervical cancer treatment [36]. The disruption of the immune system partly leads to the inability of HPV to be cleared in time and the progression to cervical intraepithelial neoplasia or even cervical cancer. VISTA blocks the response of T cells to tumor antigens. It inhibits the differentiation of T cells to Treg cells, which reduces Foxp3 protein, causing the body to develop immune tolerance to TCs, leading to the immune escape of cancer cells.

Currently, there are fewer studies on VISTA in cervical cancer. Li et al. found differences in VISTA expression in 130 cervical cancer specimens in cervical chronic inflammation, cervical intraepithelial neoplasia, and cervical cancer. There was a correlation between VISTA and cervical cancer pathological stage, and VISTA expression was higher in patients with stage II cervical cancer [37]. The risk of death with positive VISTA expression was as high as 3.184 times that of VISTA negative, suggesting that VISTA is closely associated with the development of cervical cancer. Therefore, VISTA can be used as an auxiliary indicator for cervical cancer screening, preoperative disease severity assessment, and postoperative treatment outcome monitoring.

Kuang and He systematically researched the expression of VISTA in cervical cancer by using immunohistochemistry on 104 cervical cancer specimens [38]. It was found that the high expression of VISTA on ICs was 43.27%, correlated with advanced cervical cancer and lymph node metastasis, and greatly affected the median survival of cervical cancer patients. These suggest that VISTA is a potential predictor of cervical cancer progression. Therefore, VISTA could be a candidate biomarker for cervical cancer progression and lymph node status. In addition, cervical cancer is extensively infiltrated by ICs because of long-term HPV infection. VISTA could be used in the future as a potential immunotherapy target for cervical cancer to reduce “immune escape” by treating cervical cancer patients alone or in combination with other immunotherapy strategies.

### 4.3 Endometrial cancer and VISTA

Endometrial cancer is a common malignant tumor of the female reproductive tract, and its incidence has been increasing yearly in recent years, with an average annual increase of 1.9% [39]. Although immunotherapy based on immune checkpoint inhibitors such as CTLA-4 and PD-1/PD-L1 has significantly improved the objective remission rate and overall survival of patients with advanced malignancies, there are problems such as low efficiency of monotherapy, more immune-related adverse effects, and limited benefit audience [40–42]. A retrospective analysis showed that VISTA expression was present in all endometrial cancer specimens under regulating promoter methylation status and that VISTA expressing in TCs inhibited CTLs against antigen-specific cytolysis and significantly suppressed T cell proliferation. In contrast, T cell proliferation and secretion of cytokines (IFN-γ, TNF-α) were restored when silencing the expression of VISTA [30].

Zong et al. studied 839 endometrial cancer patient tissues and found that 76.6% of ICs and 6.8% of TCs expressed VISTA, and that IC VISTA positivity was more common in FIGO stage I–III, PD-L1-positive, and DNA polymerase epsilon mutated (POLEmut) and mismatch repair-deficient types of endometrial cancer, and also positively correlated with recurrence-free survival in endometrial cancer patients [43]. VISTA is a predictor of improved prognosis independent of FIGO stage, molecular subtype, and mismatch repair functional status, and predicts improved survival in patients with endometrial cancer. However, the regulatory mechanism of VISTA in endometrial cancer is still unclear, and further studies are needed to enlighten the immunomodulatory mechanism of VISTA, as well as the prognostic impact and mechanism of anti-VISTA antibodies, to verify that VISTA can be used as a new biomarker and provide a new direction for immunotherapy of endometrial cancer.

## 5 Immunotherapy study of VISTA

Immunotherapy research on VISTA is currently underway, and several VISTA inhibitors and related antibodies are in the research phase. CA-170 is an oral small molecule antagonist targeting PD-L1 and VISTA that induces T cell proliferation and INF-γ production. *In vitro* studies have shown that CA-170 presents antagonistic effects on the PD-1/PD-L1/VISTA signaling pathway, promoting tumor infiltration and peripheral T-cell viability in a dose-dependent manner. In multiple tumor models *in vivo*, CA-170 also exhibited antitumor effects similar to those of anti-PD-1 or anti-VISTA antibodies [44]. Curis et al. investigated the efficacy of CA-170 in the treatment of patients with advanced solid tumors and lymphomas (NCT02812875).

Two VISTA-inhibitory monoclonal antibodies, CI-8993 and W0180, are currently entering clinical trials to study the therapeutic efficacy of solid tumors. Curis initiated CI-8993 in 2020 in a Phase I trial (NCT04475523) to evaluate the effectiveness, safety, and tolerability of CI8993 in treating patients with relapsed and refractory solid tumors. As well, CI-8993 was found to bind specifically to human-derived VISTA *in vivo*. WO180 was developed by Pierre Fabre as an IgG1 kappa-type antibody. The antibody is being tested in clinical trials for its antitumor effects and combination with PD-1 inhibitory monoclonal antibody (NCT04564417).

## 6 Conclusions

VISTA is closely associated with the development of gynecologic malignancies. Its unique protein structure as a “close neighbor” of B7 family PD-L1 makes it a new immunological research hotspot and promising for immunotherapy of gynecologic tumors. Research on VISTA in gynecologic tumors is continuing, and the biological role of VISTA in gynecologic tumors and the mechanisms of immune regulation in the TME still need further clarification. It is recommended to further explore the role of VISTA in the tumor immune microenvironment and patient prognosis. In the context of tumor immunotherapy, preclinical studies have shown a clear role of VISTA in anti-tumor immunity. Monoclonal antibodies and small molecules targeting human VISTA have been developed and are in early clinical trials for cancer treatment. Intensive research on VISTA is expected to improve the current problem of ineffective immunotherapy and provide new ideas for immunotherapy of gynecologic oncology patients.

## References

[1] Flies DB, Han X, Higuchi T, Zheng L, Sun J, Ye JJ, et al. Coinhibitory receptor PD-1H preferentially suppresses CD4+ T cell-mediated immunity. J Clin Invest. 2014;124(5):1966–75. 10.1172/JCI74589 PMC4001557 24743150

[2] Flies DB, Wang S, Xu H, Chen L. Cutting edge: a monoclonal antibody specific for the programmed death-1 homolog prevents graft-versus-host disease in mouse models. J Immunol Baltim Md. 1950 2011;187(4):1537–41. 10.4049/jimmunol.1100660 PMC3150865 21768399

[3] Flies DB, Higuchi T, Chen L. Mechanistic assessment of PD-1H coinhibitory receptor-induced T cell tolerance to allogeneic antigens. J Immunol Baltim Md. 1950 2015;194(11):5294–304. 10.4049/jimmunol.1402648 PMC4433880 25917101

[4] Mehta N, Maddineni S, Mathews II, Sperberg RAP, Huang P-S, Cochran JR. Structure and functional binding epitope of V-domain Ig suppressor of T cell activation. Cell Rep. 2019;28(10):2509–16.e5. 10.1016/j.celrep.2019.07.073 31484064

[5] Wang J, Wu G, Manick B, Hernandez V, Renelt M, Erickson C, et al. VSIG-3 as a ligand of VISTA inhibits human T-cell function. Immunology. 2019;156(1):74–85. 10.1111/imm.13001 PMC6283650 30220083

[6] Oliveira P, Carvalho J, Rocha S, Azevedo M, Reis I, Camilo V, et al. Dies1/VISTA expression loss is a recurrent event in gastric cancer due to epigenetic regulation. Sci Rep. 2016;6:34860. 10.1038/srep34860 PMC5056517 27721458

[7] Xu W, Hiếu T, Malarkannan S, Wang L. The structure, expression, and multifaceted role of immune-checkpoint protein VISTA as a critical regulator of anti-tumor immunity, autoimmunity, and inflammation. Cell Mol Immunol. 2018;15(5):438–46. 10.1038/cmi.2017.148 PMC6068175 29375120

[8] Bharaj P, Chahar HS, Alozie OK, Rodarte L, Bansal A, Goepfert PA, et al. Characterization of programmed death-1 homologue-1 (PD-1H) expression and function in normal and HIV infected individuals. PLoS One. 2014;9(10):e109103. 10.1371/journal.pone.0109103 PMC4184823 25279955

[9] Borggrewe M, Grit C, Den Dunnen WFA, Burm SM, Bajramovic JJ, Noelle RJ, et al. VISTA expression by microglia decreases during inflammation and is differentially regulated in CNS diseases. Glia. 2018;66(12):2645–58. 10.1002/glia.23517 PMC6585704 30306644

[10] ElTanbouly MA, Croteau W, Noelle RJ, Lines JL. VISTA: a novel immunotherapy target for normalizing innate and adaptive immunity. Semin Immunol. 2019;42:101308. 10.1016/j.smim.2019.101308 PMC7233310 31604531

[11] Wang L, Rubinstein R, Lines JL, Wasiuk A, Ahonen C, Guo Y, et al. VISTA, a novel mouse Ig superfamily ligand that negatively regulates T cell responses. J Exp Med. 2011;208(3):577–92. 10.1084/jem.20100619 PMC3058578 21383057

[12] Wang G, Tai R, Wu Y, Yang S, Wang J, Yu X, et al. The expression and immunoregulation of immune checkpoint molecule VISTA in autoimmune diseases and cancers. Cytokine Growth Factor Rev. 2020;52:1–14. 10.1016/j.cytogfr.2020.02.002 32057701

[13] Pagliuca S, Gurnari C, Kewan T, Bahaj W, Zhang K, Mori M, et al. Transcriptomic profile identifies early signatures of immunoediting and a potential role for VISTA as a molecular target in acute myeloid leukemia. Blood. 2021;138:4467.

[14] Jindal V. Immunotherapy: a glimmer of hope for metastatic prostate cancer. Chin Clin Oncol. 2018;7(6):61. 10.21037/cco.2018.02.01 29860848

[15] Choi JW, Kim YJ, Yun KA, Won CH, Lee MW, Choi JH, et al. The prognostic significance of VISTA and CD33-positive myeloid cells in cutaneous melanoma and their relationship with PD-1 expression. Sci Rep. 2020;10(1):14372. 10.1038/s41598-020-71216-2 PMC7462859 32873829

[16] Shrestha R, Prithviraj P, Anaka M, Bridle KR, Crawford DHG, Dhungel B, et al. Monitoring immune checkpoint regulators as predictive biomarkers in hepatocellular carcinoma. Front Oncol. 2018;8:269. 10.3389/fonc.2018.00269 PMC6053505 30057891

[17] Zong L, Zhang M, Wang W, Wan X, Yang J, Xiang Y. PD-L1, B7-H3 and VISTA are highly expressed in gestational trophoblastic neoplasia. Histopathology. 2019;75(3):421–30. 10.1111/his.13882 31013360

[18] Kang J, Pilones KA, Daviaud C, Kraynak J, Rodriguez-Ruiz ME, Demaria S, et al. VISTA blockade immunotherapy in a MULTI-modal approach to triple negative breast cancer (TNBC) in MICE and IMPACT on microbiome. Int J Radiat Oncol. 2019;105:S88–9.

[19] Kuklinski LF, Yan S, Li Z, Fisher JL, Cheng C, Noelle RJ, et al. VISTA expression on tumor-infiltrating inflammatory cells in primary cutaneous melanoma correlates with poor disease-specific survival. Cancer Immunol Immunother J. 2018;67(7):1113–21. 10.1007/s00262-018-2169-1 PMC11028124 29737375

[20] Deng J, Li J, Sarde A, Lines JL, Lee Y-C, Qian DC, et al. Hypoxia-induced VISTA promotes the suppressive function of myeloid-derived suppressor cells in the tumor microenvironment. Cancer Immunol Res. 2019;7(7):1079–90. 10.1158/2326-6066.CIR-18-0507 PMC6606337 31088847

[21] Blando J, Sharma A, Higa MG, Zhao H, Vence L, Yadav SS, et al. Comparison of immune infiltrates in melanoma and pancreatic cancer highlights VISTA as a potential target in pancreatic cancer. Proc Natl Acad Sci U S A. 2019;116(5):1692–7. 10.1073/pnas.1811067116 PMC6358697 30635425

[22] Liu J, Yuan Y, Chen W, Putra J, Suriawinata AA, Schenk AD, et al. Immune-checkpoint proteins VISTA and PD-1 nonredundantly regulate murine T-cell responses. Proc Natl Acad Sci U S A. 2015;112(21):6682–7. 10.1073/pnas.1420370112 PMC4450438 25964334

[23] Shahbaz S, Bozorgmehr N, Koleva P, Namdar A, Jovel J, Fava RA, et al. CD71+ VISTA+ erythroid cells promote the development and function of regulatory T cells through TGF-β. PLoS Biol. 2018;16(12):e2006649. 10.1371/journal.pbio.2006649 PMC6310287 30550561

[24] Wang L, Jia B, Claxton DF, Ehmann WC, Rybka WB, Mineishi S, et al. VISTA is highly expressed on MDSCs and mediates an inhibition of T cell response in patients with AML. Oncoimmunology. 2018;7(9):e1469594. 10.1080/2162402X.2018.1469594 PMC6140587 30228937

[25] Sung H, Ferlay J, Siegel RL, Laversanne M, Soerjomataram I, Jemal A, et al. Global cancer statistics 2020: GLOBOCAN estimates of incidence and mortality worldwide for 36 cancers in 185 countries. CA Cancer J Clin. 2021;71(3):209–49. 10.3322/caac.21660 33538338

[26] Buechel M, Herzog TJ, Westin SN, Coleman RL, Monk BJ, Moore KN. Treatment of patients with recurrent epithelial ovarian cancer for whom platinum is still an option. Ann Oncol. 2019;30(5):721–32. 10.1093/annonc/mdz104 PMC8887593 30887020

[27] Pujade-Lauraine E, Fujiwara K, Dychter SS, Devgan G, Monk BJ. Avelumab (anti-PD-L1) in platinum-resistant/refractory ovarian cancer: JAVELIN ovarian 200 phase III study design. Future Oncol Lond Engl. 2018;14(21):2103–13. 10.2217/fon-2018-0070 29584456

[28] Disis ML, Taylor MH, Kelly K, Beck JT, Gordon M, Moore KM, et al. Efficacy and safety of avelumab for patients with recurrent or refractory ovarian cancer: phase 1b results from the JAVELIN solid tumor trial. JAMA Oncol. 2019;5(3):393–401. 10.1001/jamaoncol.2018.6258 PMC6439837 30676622

[29] Zamarin D, Burger RA, Sill MW, Powell DJ, Lankes HA, Feldman MD, et al. Randomized phase II trial of nivolumab versus nivolumab and ipilimumab for recurrent or persistent ovarian cancer: an NRG oncology study. J Clin Oncol J Am Soc Clin Oncol. 2020;38(16):1814–23. 10.1200/JCO.19.02059 PMC7255977 32275468

[30] Mulati K, Hamanishi J, Matsumura N, Chamoto K, Abiko K, Baba T, et al. VISTA expressed in tumour cells regulates T cell function. Br J Cancer. 2019;120(1):115–27. 10.1038/s41416-018-0313-5 PMC6325144 30382166

[31] Liao H, Zhu H, Liu S, Wang H. Expression of V-domain immunoglobulin suppressor of T cell activation is associated with the advanced stage and presence of lymph node metastasis in ovarian cancer. Oncol Lett. 2018;16(3):3465–72. 10.3892/ol.2018.9059 PMC6096210 30127950

[32] Zong L, Zhou Y, Zhang M, Chen J, Xiang Y. VISTA expression is associated with a favorable prognosis in patients with high-grade serous ovarian cancer. Cancer Immunol Immunother. 2020;69(1):33–42. 10.1007/s00262-019-02434-5 PMC6949319 31781843

[33] Enokida T, Moreira A, Bhardwaj N. Vaccines for immunoprevention of cancer. J Clin Invest. 2021;131(9):146956. 10.1172/JCI146956 PMC8087198 33938446

[34] Orbegoso C, Murali K, Banerjee S. The current status of immunotherapy for cervical cancer. Rep Pract Oncol Radiother. 2018;23(6):580–8. 10.1016/j.rpor.2018.05.001 PMC6277269 30534022

[35] Sherer MV, Kotha NV, Williamson C, Mayadev J. Advances in immunotherapy for cervical cancer: recent developments and future directions. Int J Gynecol Cancer. 2022;32(3):281–7. 10.1136/ijgc-2021-002492 35256414

[36] Yu MC, Austin RM, Lin JF, Beck TL, Beriwal S, Comerci JT, et al. The diagnostic utility of HR-HPV as a predictor of cervical cancer recurrence. Gynecol Oncol. 2014;133:64.

[37] Li L, Xu X-T, Wang L-L, Qin S-B, Zhou J-Y. Expression and clinicopathological significance of Foxp3 and VISTA in cervical cancer. Am J Transl Res. 2021;13(9):10428–38. PMC8507058 34650712

[38] Kuang L, He Y. Potential value of V-domain Ig suppressor of T-cell activation for assessing prognosis in cervical cancer and as a target for therapy. Int J Clin Exp Pathol. 2020;13(1):26–37. PMC7013368 32055269

[39] Liu L, Habeshian TS, Zhang J, Peeri NC, Du M, De Vivo I, et al. Differential trends in rising endometrial cancer incidence by age and race and ethnicity. JNCI Cancer Spectr. 2023;7(1):pkad001. 10.1093/jncics/pkad001 PMC9904185 36625534

[40] Fontenot VE, Tewari K. The current status of immunotherapy in the treatment of primary advanced and recurrent endometrial cancer. Curr Opin Obstet Gynecol. 2023;35(1):34–42. 10.1097/GCO.0000000000000839 36595647

[41] Zheng M, Hu Y, Gou R, Li S, Nie X, Li X, et al. Development of a seven-gene tumor immune microenvironment prognostic signature for high-risk grade III endometrial cancer. Mol Ther – Oncolytics. 2021;22:294–306. 10.1016/j.omto.2021.07.002 PMC8426172 34553020

[42] Amarin JZ, Mansour R, Al-Ghnimat S, Al-Hussaini M. Differential characteristics and prognosis of PD-L1-positive endometrial carcinomas: a retrospective chart review. Life. 2021;11(10):1047. 10.3390/life11101047 PMC8540374 34685418

[43] Zong L, Mo S, Sun Z, Lu Z, Yu S, Chen J, et al. Analysis of the immune checkpoint V-domain Ig-containing suppressor of T-cell activation (VISTA) in endometrial cancer. Mod Pathol. 2022;35(2):266–73. 10.1038/s41379-021-00901-y 34493823

[44] Li K, Tian H. Development of small-molecule immune checkpoint inhibitors of PD-1/PD-L1 as a new therapeutic strategy for tumour immunotherapy. J Drug Target. 2019;27(3):244–56. 10.1080/1061186X.2018.1440400 29448849

